# Medication choice and psychosis Hospital readmissions: A two-year comparative study

**DOI:** 10.1192/j.eurpsy.2024.600

**Published:** 2024-08-27

**Authors:** O. Martin-Santiago, M. Calvo-Valcarcel, P. Martinez.Gimeno, C. Alario-Ruiz, B. Arribas-Simon

**Affiliations:** Hospital Clinico Universitario, Valladolid, Spain

## Abstract

**Introduction:**

Hospital readmissions in psychosis are a critical concern, with medication choice playing a vital role. Oral antipsychotics, though common, rely on patient adherence and can lead to relapses if not followed. Long-acting injectable atypical antipsychotics (LAIAs) provide an alternative, ensuring consistent medication release and reducing relapse risk due to missed doses. Studies indicate that LAIAs result in fewer readmissions due to improved adherence. Tailoring treatment to individual needs is essential. Medication choice significantly influences hospital readmission prevention in psychosis. LAIAs, which could offer greater adherence to treatment and symptom control, present a promising option. Individualized treatment decisions are a priority for long-term recovery.

**Objectives:**

This study aimed to compare the hospital readmission rates within two years post-discharge among two groups of patients diagnosed with schizophrenia and other psychotic disorders who received either oral antipsychotic treatment or LAIAs.

**Methods:**

We collected sociodemographic and hospitalization data from 155 patients, 90 receiving oral antipsychotics and 65 receiving LAIAs, following their discharge from a psychiatric unit.

**Results:**

There were 90 patients in the oral treatment group, and 65 in the LAIA group, with 67.6% receiving paliperidone and 26.1% receiving aripiprazole. There were no significant differences in age or gender between the two groups. However, patients in the LAIA group had *longer stays in the hospital* (M=14.7; SD=10.2 vs M=11.1; SD=6.4; t_(153)_=2.67; p<.01) and a higher number of prior admissions (M=3.2; SD=3.7 vs M=1.3; SD=3.5; t_(153)_=2.41; p<.01) compared to the oral antipsychotic group. Additionally, a higher percentage of patients in the LAIA group were diagnosed with schizophrenia (60%) compared to the oral antipsychotic group (24%) (*X^2^*_(1, N = 155)_= 20.4, p<.01). After two years, readmission rates were 66.6% for the oral antipsychotic group and 61.5% for the LAIA group (*X^2^*_(1, N = 155)_= 8.5, p > .05). However, the time to readmission was shorter for patients on oral antipsychotics (M=172.4; SD=162.0) compared to those on LAIAs (M=326.2; SD=211.4; t_(153)_=3.05; p<.01). Notably, 86.6% of patients on oral antipsychotics were readmitted within the first year, while only 52% of those on LAIAs experienced readmission during the same period (*X^2^*_(1, N = 155)_= 8.5, p = .001).

**Conclusions:**

Long-acting injectable antipsychotics (LAIAs) appear to reduce hospital readmissions, with a more pronounced effect in the first few months post-discharge. However, after two years, the readmission rates between LAIAs and oral antipsychotics become comparable. This data suggests that while LAIAs may reduce early readmissions, their long-term effectiveness is on par with oral antipsychotics.

**Disclosure of Interest:**

None Declared

